# The dynamic of tuberculosis case finding in the era of the public–private mix strategy for tuberculosis control in Central Java, Indonesia

**DOI:** 10.1080/16549716.2017.1353777

**Published:** 2017-08-02

**Authors:** Reviono Reviono, Wahyu Setianingsih, Kusmadewi Eka Damayanti, Ratna Ekasari

**Affiliations:** ^a^ Faculty of Medicine, Universitas Sebelas Maret, Surakarta, Indonesia; ^b^ Dr Moewardi Hospital, Surakarta, Central Java, Indonesia; ^c^ Office of Health of Central Java Province, Semarang, Central Java, Indonesia; ^d^ KNCV (Koninklijke Nederlandse Centrale Vereniging tot Bestrijding der Tuberculose) Central Java, Indonesia

**Keywords:** Case detection rate, public–private mix, tuberculosis, monitoring, evaluation

## Abstract

**Background**: The public–private mix (PPM) strategy has strengthened tuberculosis care and control in many countries. Indonesia, a country with a high tuberculosis burden, has a low tuberculosis case detection rate (CDR), despite PPM implementation in 2003. The PPM in Indonesia involves primary healthcare centers, hospitals, and specialized chest clinics. The long-term impact of the strategy is unknown.

**Objective:** We aimed to explore the case detection achievements of the tuberculosis program since PPM implementation in Central Java in 2003.

**Methods**: This retrospective cohort study covered the period 1 January 2000 to 31 December 2014. The data from tuberculosis patients treated in all health facilities in Central Java implementing directly observed treatment short-course, recorded via a standardized form, were analyzed after being validated by the Office of Health of Central Java Province. We evaluated the CDR, case notification rate, and total number of cases, using linear regression to analyze the temporal trends of those indicators in the phases of PPM implementation.

**Results**: The CDR increased during the initial phase (2000–2005), decreased during the mid-phase (2006–2009), and increased slightly during the late phase (2010–2014), ranging from 13 to 61.72. These trends were observed despite a steady increase in the number of participating healthcare facilities. The regression analysis showed that the CDR of referral institutions contributed the most to the total CDR of Central Java Province. Many of the smear-negative tuberculosis cases recorded at primary healthcare centers may have been smear positive; this probable misclassification could have been partially avoided if more specific and sensitive diagnostic tools were available.

**Conclusions**: The CDR remains below the national target (70%). Early awareness of a negative trend in certain program indicators is important to ensure program sustainability. Careful observation of the indicator pattern will secure the long-term success of the program.

## Background

The Public–Private Mix (PPM) strategy of the tuberculosis (TB) control program by the World Health Organization (WHO) was introduced in 2006 [1]. It is the fourth of six components in the Stop TB Strategy of 2006 and is the second pillar of the End TB Strategy [2,3]. It has been proven in many countries that PPM strengthens global TB care and control [2,4–7]. Some of the positive impacts of PPM implementation include an increased rate of notification of new acid-fast bacilli (AFB) smear-positive pulmonary TB cases [2,4,8], improved cost effectiveness [7], and increased quality of sputum examination [9].

Indonesia is a country with a high burden of TB cases, with the second highest incidence of TB cases reported worldwide in 2015 [2]. WHO has set the three assessment targets, namely a decline in TB incidence, and a 50% reduction in both the prevalence and mortality rate by 2015 compared to 1990. However, Indonesia has fulfilled only one of them, which is the decline in TB incidence. Another setback is the low tuberculosis case detection rate (CDR) as reported by the WHO in 2015 [2]. The CDR is determined by the number of new AFB-positive smears [1], and Indonesia still has a low detection rate for AFB-positive smears, likely due to the low sensitivity of the current microbiological detection tools. CDR is a strategic indicator because the achievement of a 70% CDR, followed by cure rate at 85% or higher, will decrease the annual TB incidence rate by 7–12% [1,6]. Indonesia implemented the PPM in 2003 by recruiting public and private hospitals; the system is known as Hospital DOTS Linkage (HDL). The HDL pilot projects were conducted in four provinces (Yogyakarta, South Sumatra, West Sumatra, and Bali) during the period 2000–2005. Because of these pilot projects, PPM was scaled up at the national level in 2003 [10].

Central Java, with a population of 33,522,663, is one of the biggest provinces in Indonesia [11], thus, the targets achieved in Central Java influence the national achievements. Central Java has implemented the PPM since 2003, recruiting primary healthcare centers (Puskesmas), public and private hospitals, and specialized chest clinics. The HDL activities include strengthening case management networks and patient referrals, laboratory quality assurance, supervision, and monitoring and evaluation. Hospitals recruited to the HDL are those committed to implementing the directly observed treatment short course (DOTS) strategy. Another requirement is that all hospital staff – medical and administrative– must have undergone training in the implementation of the DOTS strategy [12]. The program funding, evaluation, and support systems such as medical and administrative staff are likely to be the factors that heavily influence the success of the program, including the PPM program in Indonesia.

The PPM network was established at a provincial level in 2002. The network elected a coordinating committee, which monitored the implementation of the PPM strategy. In 2003, a hospital-level coordinating committee was established in each hospital and chest clinic. The DOTS units were set up in each healthcare center and a specific facility was built in each unit. The DOTS strategy was implemented by following the standard operational procedure of registering and treating the patients, allocating drugs, and referring patients for follow up or for more extensive treatment [10].

PPM implementation has led to strengthening of TB care and control, especially in terms of increasing TB case detection and notification in almost all regions, but these results are based on data collected over no more than five years [4,5,8,12]. Data monitoring over a longer time period is needed to evaluate the long-term impact and sustainability of the PPM strategy. This study aimed to explore the changes in target achievement over the past 15 years (2000–2014) during which the PPM program has been operational in Central Java, focusing on TB case detection.

## Methods

### Study area and setting

Subjects were TB patients treated in all DOTS-implementing health facilities in Central Java Province between 1 January 2000 and 31 December 2014. Central Java is a dense province with 33,522,663 people residing in an area of 32,544.12 km^2^. It is divided into 29 regencies and 6 municipalities. The DOTS program for TB eradication was established in 1995, while the PPM strategy was implemented in 2003 [11], following program preparation in 2002 [13]. The preparation stage was comprised of: obtaining commitment from the government and healthcare service providers; training of trainers for hospital and clinic staff, including doctors, nurses, laboratory technicians, pharmacists, medical record personnel, and health promoters; on-the-job follow-up training; and developing the PPM network.

The DOTS Strategy was implemented first in the primary healthcare centers. Then, in 2003, the number of specialized chest clinics was increased. One year later, hospitals joined the PPM after sufficient preparation and adjustments. Hospitals played a strategic role in PPM implementation, especially in treating patients with TB.

A standardized form was used to register TB cases. Patients were monitored by treatment observers who regularly received information updates and health education from the staff. Medication was issued weekly or monthly and was collected either by the treatment observer or the patient.

The referral system for treatment and follow-up was based on four options: (1) Both the diagnosis and initiation of TB treatment in hospital, followed by referral to a primary healthcare center for Directly Observed Therapy, with subsequent clinical follow-up in a hospital; (2) Similar to that of the former, but with clinical follow-up at a healthcare center, not a hospital; (3) Both the diagnosis of, and directly observed treatment for, TB occurring in hospital, followed by referral to a health center during the course of treatment; or (4) Diagnosis of TB and subsequent hospitalization for the full course of directly observed treatment.

Intensive monitoring was conducted periodically to review case detection and treatment, manage the collected data, cross-check the laboratory findings and referral status, and trace defaulters. The strategies and referral system were discussed and problems were listed as topics to be discussed at higher-level meetings. Besides constructing intra-hospital networks, inter-hospital networks were also established to ensure the successful application of the program.

### Study design

This was a retrospective cohort study using reported data from healthcare services implementing the DOTS strategy. It included quarterly data from the primary healthcare centers and referral institutions (hospitals and chest clinics) collected over the period 2000–2014. The data were extracted from the TB DOTS standard report forms from each regency and District Office of Health. The following data to be analyzed were extracted and compiled in a research database: number of new AFB smear-positive and AFB smear-negative TB cases; number of TB relapse cases; number of cases of extra-pulmonary TB; and the outcome of treatment. The entered data were verified by the researchers (in terms of characteristics, types of cases, and outcomes) to construct a final database. The final database was then expedited to the Central Java Province Office of Health to be verified by the authorized officer; once verified, the dataset was then analyzed.

### Variables

The database was analyzed for epidemiologic indicators of TB and TB treatment, such as the CDR (%), case notification rate (CNR, %), total number of TB cases, and AFB-positive and AFB-negative cases. An AFB smear-positive pulmonary TB case was defined as a patient having at least two initial direct sputum smear microscopy examinations with AFB positivity, or one AFB-positive direct sputum smear microscopy examination and radiographic abnormalities consistent with active pulmonary TB, as determined by a clinician. AFB smear-positive cases are the most infectious and thus of the highest priority from a public health perspective. An AFB smear-negative case of pulmonary TB was defined as a patient with pulmonary TB not meeting the above-mentioned criteria for smear-positive disease. Diagnostic criteria include at least two AFB-negative sputum smear examinations, radiographic abnormalities consistent with active pulmonary TB, no response to a course of broad-spectrum antibiotics (except patients for whom there is either laboratory confirmation, or strong clinical evidence of human immunodeficiency virus infection), and a decision by a clinician to treat with a full course of anti-TB chemotherapy. Patients with a positive culture but AFB-negative sputum examinations are also classified as smear-negative case of pulmonary TB [14]. The total number of TB cases was the sum of all AFB smear-positive, AFB smear-negative, and extra-pulmonary TB cases.

The CDR was calculated by dividing the number of new AFB smear-positive cases notified in one year by the annual incidence from notification data, and estimates of incidence (estimates based on the national target of a CDR of at least 70%). The estimation used for national incidence was 107 per 100,000 population as published by the Ministry of Health. The CNR was derived from the definition of incidence of disease set by the WHO, calculated from the number of new cases of TB disease (all forms) per 100,000 population, per year [1].

### Analysis

The data were depicted in graphs and tables. The pattern of CDR and the independent variables such as CDR of referral centers, the number of healthcare facilities, and the AFB-smear positive cases, were analyzed using linear regression methods, stratified by time-period. Time was divided into three periods, according to the phases of PPM implementation, as follows: an initial phase (2003–2005), mid phase (2006–2009), and late phase (2010–2014). The units of analysis in this study were: the annual CDR; the annual CNR; number of TB cases; AFB smear-positive cases; AFB smear-negative cases; and the number of healthcare facilities (including primary healthcare facilities, hospitals, and specialized chest clinics) involved each year.

### Ethical considerations

Ethical approval was granted by the Ethics Committee of Dr. Moewardi Hospital/Faculty of Medicine, Sebelas Maret University before the research started (ethical clearance no. 835/IX/HREC/2016). Confidentiality and anonymity were obtained by assigning codes to each regency once the data had been validated by the officer of the Provincial Office of Health. Coding also ensured the confidentiality of the officer who collected and validated the data. The researcher did not have access to the codes until the final data analysis had been completed.

## Results

**Figure 1.**
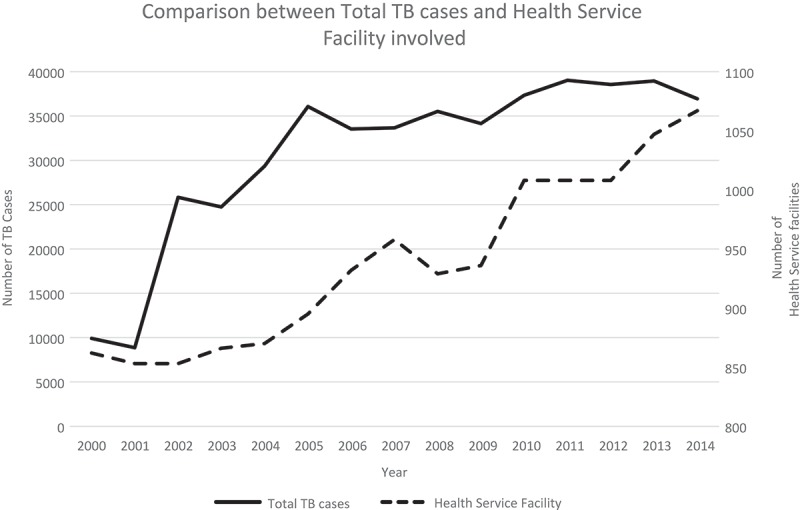
Comparison between total number of tuberculosis (TB) cases and number of healthcare facilities (including primary healthcare facilities, hospitals, and specialized lung clinics) in the TB Control and Care program. The Public–Private Mix (PPM) strategy was implemented in 2003.

At the beginning of the program in 2003, PPM involved primary healthcare centers and specialized chest clinics. In total, 866 healthcare facilities – 855 primary healthcare centers and 11 chest clinics – were involved in the PPM program in 2003. By 2014, this number had grown to 1067 healthcare facilities: 875 primary healthcare centers, 11 chest clinics, and 171 hospitals. TB case detection of all types of TB (AFB smear-positive, AFB smear-negative, and extra-pulmonary TB cases) increased from 24,737 cases in 2003 to 36,947 cases in 2014 with fluctuations in the total case count in the intervening period. In the early phase of PPM implementation, the increase in the number of healthcare facilities involved was accompanied by an incremental rise in the total number of TB cases, but this plateaued after 2012 (Figure 1).

**Figure 2.**
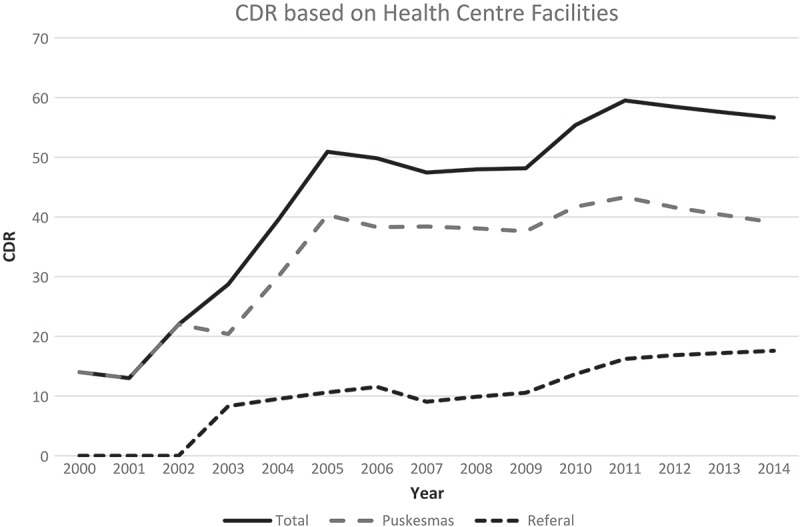
Pattern of the case detection rate (CDR) based on health facility, and the total CDR of the province.

The CDR increased during the initial phase of PPM implementation. During the mid-phase (2006–2009), the CDR decreased slightly, with slight variation year-on-year, as demonstrated by the gradual slope during this period (Figure 2). The CDR improved after 2009 but again decreased in the period 2011–2014. Based on the regression analysis results, during the early phase the CDR of referral centers and the number of healthcare facilities involved contributed to the total CDR, although this was not statistically significant (R^2^ = 90.4%; β_CDR Referral_ = 1.56, p-value = 0.173; β_Health facility_ = 0.432, p-value = 0.239). The CDR of referral centers also contributed to the CDR of the province in the mid-phase (R^2^ = 88,4%; β_CDR Referral_ = 0.909, p-value = 0.058) and in the late phase (2010–2014) (R^2^ = 92.5%; β_CDR Referral_ = −0.87, p-value = 0.148), and also to the number of AFB smear-positive cases detected in the late phase (β_AFB(+)_ = 0.432, p-value = 0.051). The referral institutions seem to play a role in case detection; therefore, CDR was increased in the first phase. However, in the mid- and late-phase, the contribution of referral CDR became less important in the achievement of total CDR. The possible explanation for the CDR increase in the late-phase may likely have been the result of the program evaluation held in 2010.

**Figure 3.**
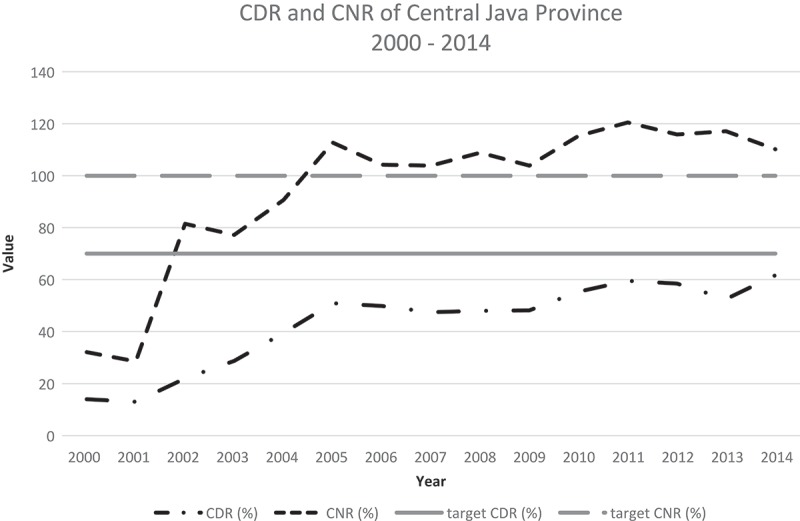
Comparison of the annual case notification rate (CNR) and case detection rate (CDR) in the period 2000–2014 with the CDR target of 70% set by the TB Control and Care program.

**Figure 4.**
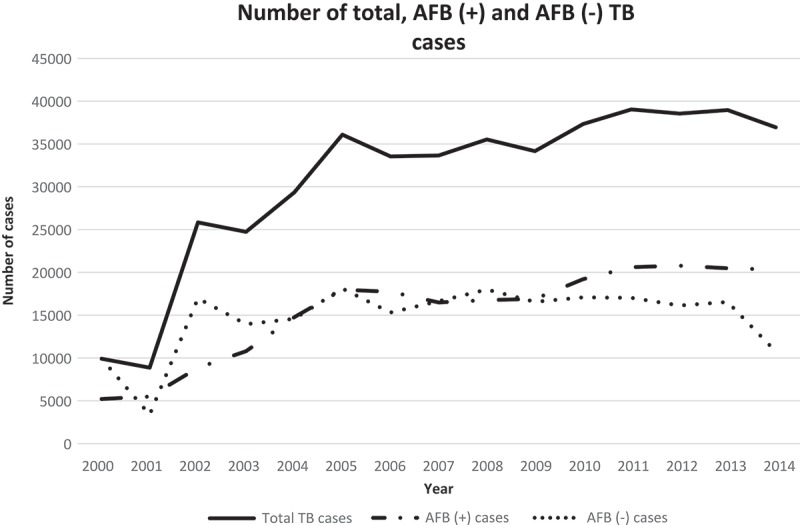
Comparison of the number of total tuberculosis (TB) cases, acid-fast bacilli (AFB) smear-positive TB cases, and AFB smear-negative TB cases during the period 2000–2014.

The government set the CDR target at 70% and the CNR target at 100%. Since 2005, the total number of TB cases has remained >30,000 per annum. Figure 3 shows that the CNR was persistently >100%. However, the CDR was never above the 70% target. To reach the CDR target of 70%, the minimum case detection of AFB smear-positive TB cases should reach at least 25,000 annually; this has not happened in the last five years (Figure 4). However, the annual number of AFB smear-negative TB cases was high (>15,000) each year except 2014.

## Discussion

### The dynamic of PPM implementation

The aim of this study was to explore the changes in the target achievement program, especially regarding TB case detection in the 15 years before and during PPM implementation in Central Java. This study found that the CDR was very low before the implementation of PPM (2000–2003); however, it increased during the implementation (Figure 2). However, the CDR did not reach the set target, and as such TB case detection in Central Java remains a significant concern. The other important finding of this study was the increasing number of healthcare facilities which were involved in the PPM program in Central Java (Figure 1).

A health intervention program is considered advantageous, when continuous use of its intervention components and activities results in ongoing achievement of desirable health outcomes, within the population of interest [15]. An increasing trend is usually found in the initial implementation stage of the program, but the challenge to sustain the achievements of the program remains, especially in terms of implementation in the field [16]. This research was not intended to appraise the sustainability of the PPM program; rather, it aimed to assess the achievement of several of the program’s indicators, such as the CDR and the number of AFB smear-positive cases. Our approach differed from other studies that usually present expert opinions rather than empirical inputs and outputs; we restricted our analysis to data measured in this study.

This study found that the CDR and successful case detection fluctuated for both total, and AFB smear-positive, TB cases (Figure 4). During the initial phase, there were substantial and gradual increases in both case detection, and CDR, respectively. In contrast, both indicators decreased during the mid-phase. In the late phase, there were increases in case detection and the CDR, but these increases were not as rapid as in the initial phase. The regression analysis showed that the CDR of referral centers consistently contributed to the total CDR achievement of Central Java Province, although this was not statistically significant. The number of healthcare facilities involved also influenced the CDR achievement in the early period, probably due to the vigorous networking that occurred during the early period of the program (Figure 1). In the late period, the number of AFB smear-positive cases contributed little to the CDR, implying that detection of more AFB smear-positive cases might increase the total CDR. Moreover, it is proposed that some cases may have been misclassified, namely, that the probability of false-negative sputum smears exists; that some patients classified as being AFB smear negative based on laboratory testing were in fact, smear-positive. This implies that there is a need for more specific and sensitive tools for diagnostic testing.

The implementation of the PPM strategy in Central Java Province focused on the number of hospitals involved, as hospitals play a strategic role, often being the first healthcare facility visited by TB patients for treatment initiation [13]. The involvement of hospitals as stakeholders in the National TB Program is referred to as HDL. The Indonesian national 2004 TB prevalence report found that most TB patients (49%) sought healthcare for TB at hospitals and specialized chest clinics, compared to private general practitioners (29%) and primary healthcare centers (21%) [17]. During the initial phase of PPM implementation, both hospitals and primary healthcare centers enthusiastically implemented the program. Before the PPM, healthcare facilities often lost patients to follow-up and had no record of the treatment outcome, indirectly failing to stop TB from spreading and increasing the threat of more infectious and drug-resistant TB cases occurring in the community. During PPM implementation, however, the various healthcare facilities were linked by the program and worked together to treat TB patients.

The process then proceeded to the mid-phase, where there was a decrease in the achievement indicators. Factors influencing this phenomenon included the cessation of funding from one funding donor – this impacted on the daily operational activities. In response, the National TB Program sought a new funding donor for the program. Rotation of hospital staff also influenced the program, resulting in the observed fluctuation in the indicators [12]. The rotation of hospital staff required an adaptation on the part of the remaining staff in terms of different program-related capabilities, different styles of work, and probably a different pace of work; adjustment to a new team may take some time.

The next period was the late phase. During this phase, the indicators improved. One factor likely related to this was that an assessment of how hospitals had implemented the TB program in Central Java Province was conducted. This assessment was performed by the Office of Health using tools established by the Ministry of Health, including assessment of training personnel, facilities, and systems regarding treatment and services for TB patients. This assessment triggered hospital managers to improve their hospital’s performance, including their TB management service. The number of hospitals involved in PPM implementation also increased, although the number was small. However, in the last two years of the late phase, there was a decrease in the indicators.

PPM implementation was dynamic and was inevitably influenced by many factors, with a particular pattern observed in each phase. Iwelunmor et al. [16] proposed that there are core components for the sustainability of an intervention program; these are: healthcare worker shortage; lack of financial leadership; and weak health systems. Lack of funding support for a program is another problem in terms of sustainability, especially in the context of health intervention programs. Shediac-Rizkallah and Bone defined successful sustainability of a program as being reached when the program is still running after discontinuation of funding support [18]. Both opportunities and threats influence a program [16]; both can be managed positively, depending on how policy actors respond to the issues raised [19].

A program evaluation mechanism is important to foresee and prevent a downwards trend of a program in order to achieve sustainability. Anticipatory actions include reassessment of program implementation, assessment of program sustainability, refresher training for the PPM staff, integrating the health insurance system in program budgeting, and collaborating with other programs, especially those with a higher budget and wider impact.

Because a downward trend in CDR was observed in primary healthcare centers in the late period, anticipatory actions should have been taken to prevent this. Assessment of program performance is an important action, as seen by the improvement at referral institutions while the assessment was being conducted. Refreshing staff skills and ability can also contribute to improving the case detection achievement in primary healthcare centers. The skill of the staff also influences the detection of AFB smear-positive cases; the rate of false negative results can increase because of poor laboratory examination skills. Thus, program assessment in primary healthcare centers and refreshing of staff skills should be conducted in anticipation of falling trends in the CDR.

### Program achievements

It has been reported that in other countries the CDR increased significantly because of successful PPM implementation. However, the WHO reported that Indonesia has still not reached the CDR target [2], despite recruiting healthcare facilities to the PPM. The present study was undertaken in response to the WHO report stating that Indonesia’s CDR achievement remains below target.

There are several reasons for the low rate of AFB smear-positive TB case detection and the low CDR. The WHO explained that one of the reasons for low achievement was under-reporting of diagnosed TB cases, especially done by the private sector [2]. All the PPM stakeholders had undertaken extensive measures to recruit greater numbers of healthcare services to participate in the PPM program. This effort was followed by extensive spread of information and training of both medical and administrative staff in terms of DOTS implementation.

The number of TB total cases and the CDR showed a steep increase during the period 2003–2005; this was followed by a decrease in values of both of these indicators. Previous studies reported that the program showed good results and increased achievement within five years post-implementation, but these studies did not follow the program beyond five years [2,4–7]. A measure of sustainability is required because the PPM strategy has been implemented from 2003. Until recently, the CDR was considered the main indicator of successful TB eradication, with the CDR target set at 70% [2]. Most countries have reached the target, however Indonesia has not [2,20]. Figure 2 shows that the CDR in Central Java Province is still <60% [11].

The number of healthcare facilities involved in the PPM program has increased every year. However, the number of cases detected did not increase proportionately, neither did the number of AFB smear-positive cases. This finding implies that successful PPM implementation is based not only on the number of healthcare facilities involved but also on other factors. In contrast, PPM implementation has improved the CNR in Central Java Province, from 77.2% in 2003 to >100% in the years thereafter, demonstrating a continuous and sustained effort in notifying cases.

As a diagnostic tool, sputum direct smear microscopy for AFBs has low sensitivity. This means that the lower the concentration of bacilli in a sputum specimen, the lower the probability of obtaining a true positive result [21]. Use of low-sensitivity diagnostic tools can result in inaccuracies in detection, increasing the probability that true AFB smear-positive cases are misclassified as being AFB smear-negative. These results are indirectly misleading, especially when the indicator is used as a basis for decision-making. To improve case detection, better diagnostic tools are required. The WHO recommends use of the Xpert MTB/RIF (Cepheid, Sunnyvale, CA, USA) for TB diagnosis [22,23]. This molecular-based tool can detect AFB-positive patients with greater precision and accuracy, applying a combination of polymerase chain reaction and fluorescence detection techniques. Therefore, it is important for the national TB program to implement the use of Xpert MTB/RIF for TB diagnosis.

The outcome of this TB control program was measured not only by CDR outcomes, but also by its success rate. A success rate target of 85% was set to maintain a high-quality program. In general, the implementation of PPM has not yet improved the AFB smear-positive case detection rate, or CDR outcomes. However, the quality of the program is still as expected from the measure of the success rate, which reached the target of 85%. Although the cure rate indicator has not yet reached the target, there is a narrow gap which will be feasible to reach with a reasonable effort. This is important because a low success rate means a low number of cured cases and a high probability of drug-resistant cases emerging.

### Strengths and limitations of the study

The limitations of this study include the limited number of variables and weak results obtained. The limited number of included variables, recorded from the secondary database only, restricted the analysis, leading to an incomplete explanation of the observed phenomena. However, this study provided quite a long array of data, covering a period of >10 years (2000–2014). This was thus a more comprehensive evaluation of the long-term effect of a program than most other studies that usually assess the first three to five years of a program.

### Public health implications

The public health implications of this study relate to the long-term observations on the dynamics of a program, particularly the TB program. This study shows that an increase in case detection is expected in the initial phase of a program, whereas a stagnant, or slower, pace of increase is experienced several years into the program. This information can assist program managers, especially those who have managed a program in a setting similar to the one discussed here, by making them aware of the temporal trends, thereby enabling them to engage in anticipatory actions to improve program outcomes and sustainability. Although the study cannot provide more detail regarding the changing trends, it does demonstrate the program dynamics over >10 years.

## Conclusion

Implementation of the PPM for TB treatment and control in Central Java Province was quite dynamic, with a rapid scale-up initially, a stagnant mid-period, and then a slight drop. It is important to observe the pattern of each phase carefully and to be aware of decreasing trends in the indicators. It is expected that, by careful observation of the pattern, an early warning system can be developed to identify an unsuccessful program. The study findings imply the need to assess the sustainability of the national TB program, especially in terms of the PPM strategy. The use of diagnostic tools with high sensitivity and specificity to detect AFB smear-positive TB cases is needed to minimize the misclassification of AFB smear-positive cases as AFB smear-negative cases.

## Paper context

The Stop TB Strategy, a means to control the global spread of TB, was launched with the vision of the world free from TB. One of its strategies is implementing collaboration between the public or government sector and the private or non-government sector. This collaboration is known as the Public–Private Mix (PPM); Indonesia has implemented it since 2003. This study aims to explore the achievement of the TB program shortly before and after the implementation of PPM.
